# Idiopathic Symmetrical Peripheral Gangrene: A Rare Clinical Entity

**DOI:** 10.7759/cureus.14447

**Published:** 2021-04-13

**Authors:** Saurabh Gaba, Arshi Syal, Yajur Arya, Monica Gupta, Amanjot Kaur

**Affiliations:** 1 General Medicine, Government Medical College and Hospital, Chandigarh, IND

**Keywords:** symmetrical peripheral gangrene, idiopathic, nose, fingers, gangrene

## Abstract

Symmetrical peripheral gangrene (SPG) is a rare disorder leading to ischemic necrosis of extremities. We present a rare case of idiopathic SPG in a 58-year-old male who did not seek any medical care, and history was elucidated only after he presented one year later for treatment of pneumonia. Extensive investigations revealed no etiology. SPG can result from a variety of conditions that cause disseminated intravascular coagulation and lead to distal hypoperfusion. The treatment is aimed at the control of the underlying disease and wound management.

## Introduction

Symmetrical peripheral gangrene (SPG) is characterized by symmetric, distal limb ischemia not associated with any specific vaso-occlusive disorder [1]. It classically occurs bilaterally and in the absence of an established thrombotic disease. Disseminated intravascular coagulation (DIC) is central to the pathogenesis of SPG, with certain hypercoagulable states presumed to play a role in disease initiation [2,3]. This knowledge is important as the management of SPG is aimed at reversal of DIC and the causative phenomenon. This can be achieved medically; however, surgical intervention is frequently required. Although treatable if detected early, SPG has high morbidity and mortality, mainly because of its association with serious conditions and the rapid spread of gangrene to proximal areas.

## Case presentation

**Figure 1:**
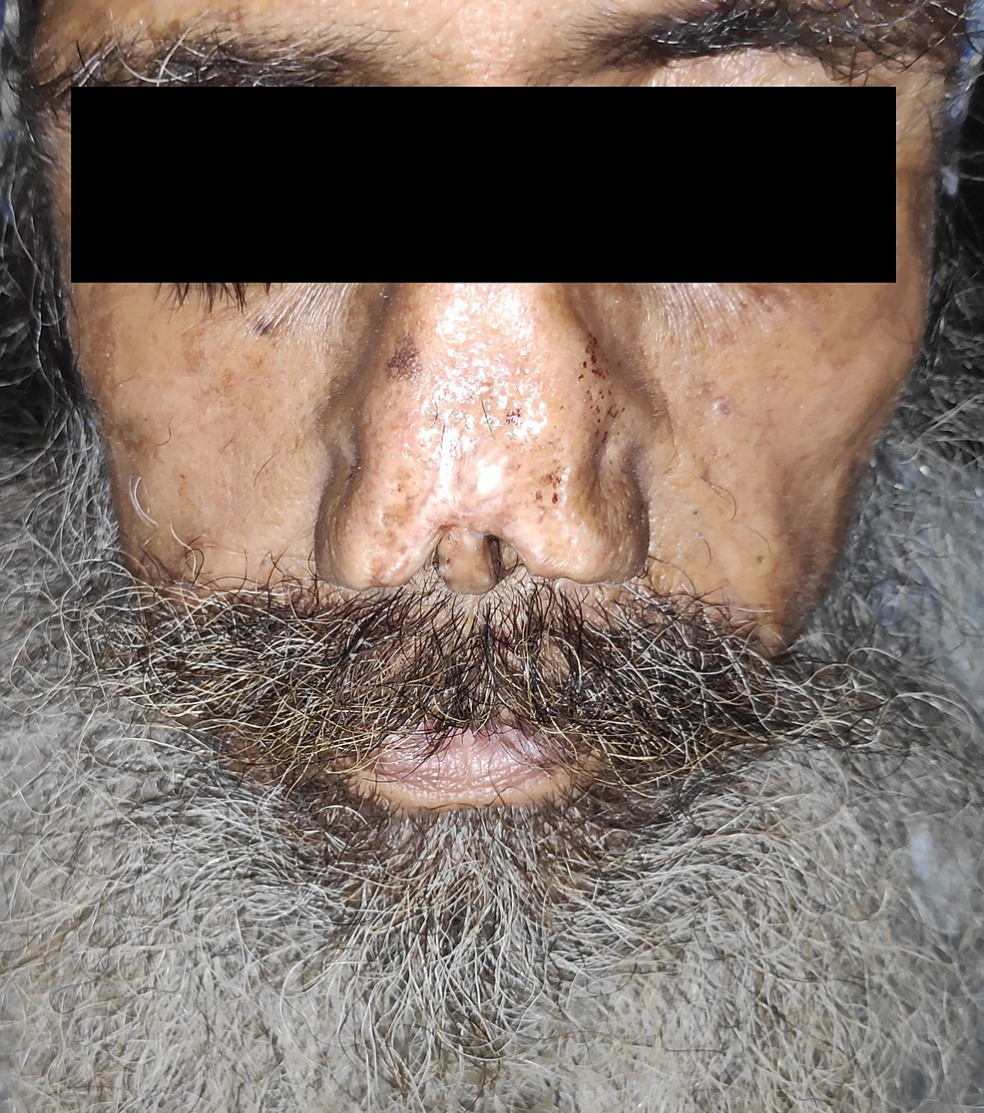
Amputated tip of the nose.

**Figure 2:**
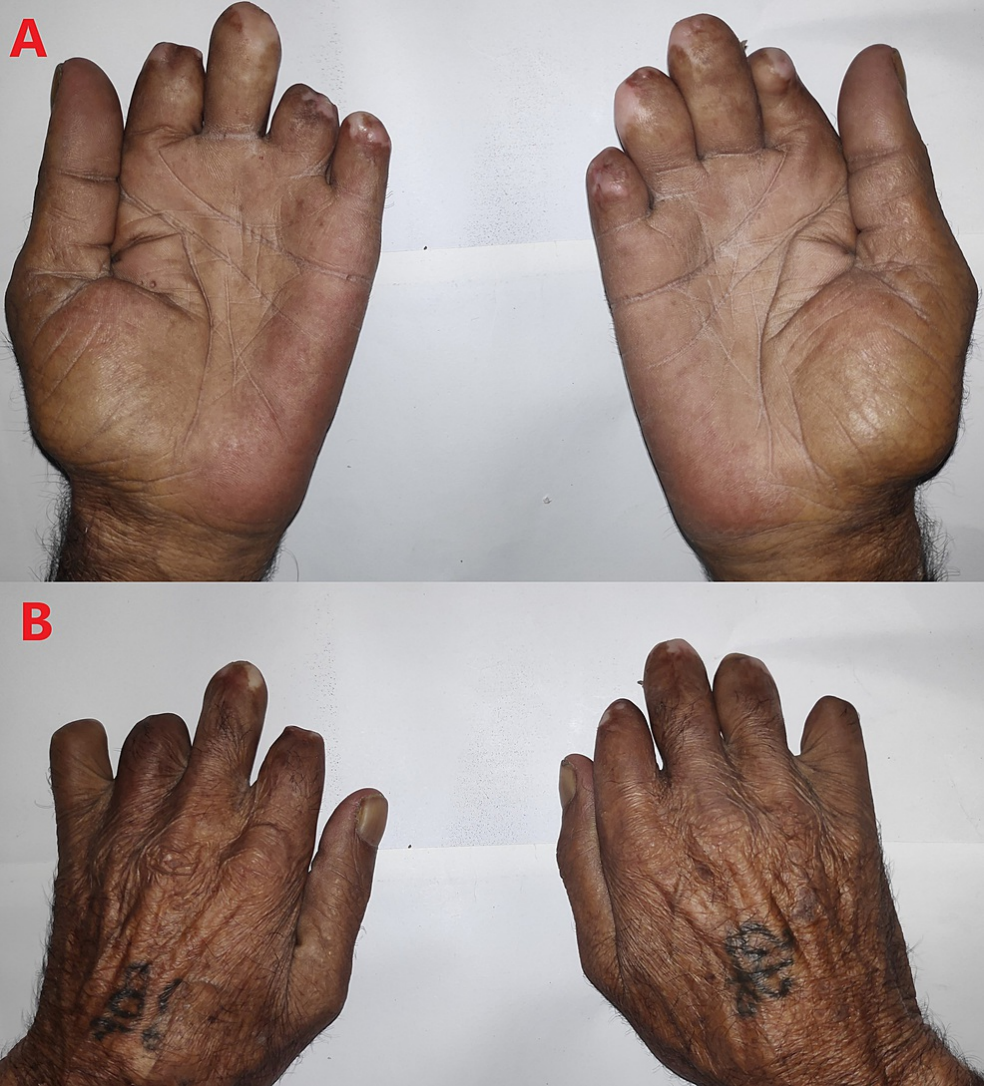
Amputated fingers of both hands (A and B) with intact thumbs.

A 58-year-old male, a farmer by profession, was admitted to the hospital for management of lobar pneumonia that responded well to treatment. A year prior to presentation, he had developed painful black discoloration of the tip of nose and fingers of both hands, excluding the thumbs (Figures 1, 2). The affected areas shriveled up and were shed off with minimal bleeding within days of onset of the symptoms. The lower limbs and ears were not affected. No medical care was sought at that time. He reported being otherwise healthy in the past and had no co-morbidities. He did not smoke and did not consume any prescription, over-the-counter, or recreational drugs.

At the time of the affliction, he did not have any history of fever, weight loss, rash, joint pains, oral or genital ulcers, limb weakness or paresthesias, chest pain, breathlessness, cough, hemoptysis, limb claudication, Raynaud’s phenomenon, hematuria, dryness of eyes or mouth, and bowel or bladder symptoms. Extensive investigations were carried out to rule out any underlying hypercoagulable states (protein C, protein S, antithrombin III, factor V Leiden mutation, homocysteine, and lipids), autoimmune disorders (antinuclear antibodies, antineutrophilic cytoplasmic antibodies, antiphospholipid antibodies, cryoglobulins, rheumatoid factor, and complement levels), malignancy (chest X-ray, ultrasound abdomen, and serum electrophoresis), and vascular disease (echocardiography and arterial doppler); however, they were all unremarkable. Based on his previous history, clinical presentation, and absence of a clear etiology on investigations, a diagnosis of idiopathic SPG was made. Despite SPG being associated with severe and potentially fatal outcomes, this patient had an innocuous clinical course, although it resulted in marked physical deformity and functional disability.

## Discussion

SPG is an infrequently reported entity characterized by microvascular ischemia and dry gangrene of the extremities (limbs, ears, and nose) [1]. Not only does this lead to life-altering repercussions even after treatment but also carries a high rate of mortality. In most cases, it is a result of DIC causing thrombotic occlusion of capillaries and downstream hypoperfusion. The etiology is diverse and includes sepsis, heart failure, shock, malignancies, hypercoagulable conditions (commonly protein C and S deficiency), myeloproliferative neoplasms which lead to hyperviscosity of the blood, various autoimmune conditions including systemic lupus erythematosus, antiphospholipid antibody syndrome, and cryoglobulinemia [4,5]. Reports in literature have also associated this entity with infections such as malaria, dengue, and meningococcemia [3,6,7]. Other causes include sickle cell anemia, cold agglutinin disease, and inotrope use. Idiopathic SPG has also been reported [1]. There is no abnormality in the large blood vessels, and the condition needs to be differentiated from other causes of gangrene such as peripheral vascular disease, thromboangitis obliterans, embolic phenomena, and Raynaud’s phenomenon. The management is specific to the cause and treatment modalities, depending on the etiology, including anticoagulation, antibiotics, immunosuppression, hemodynamic optimization, and withdrawal of the inotropes if possible [1,8]. Anecdotes are available to support the use of hyperbaric oxygen, trimethaphan for sympathetic ganglion block, topical nitroglycerine, and intravenous epoprostenol, phentolamine, and nitroprusside. [1,9] These therapies require more evidence before they can be a part of the standard treatment. Plasmapheresis and thrombolysis have not been found to be effective. Surgical management of the affected areas is often required in the form of debridement or amputation if the affected areas cannot be salvaged [8]. The cause and treatment in idiopathic SPG, as in the case presented, remain obscure, and the selective involvement of nose and fingers with autoamputation is captivating.

## Conclusions

SPG can occur as an idiopathic phenomenon. The management and both the short- and long term outcomes in such a case remain undefined. Efforts should be made to not miss any underlying disease process such as a malignancy, thrombophilia, infection, and autoimmune disease. The intriguing case presented here was brought to notice a year after the event and was characterized by a very rapid clinical course with no apparent lasting effects on health, albeit resulting in functional limitation and disfigurement.
